# Severe hypoglycemia and the risk of end stage renal disease in type 2 diabetes

**DOI:** 10.1038/s41598-021-82838-5

**Published:** 2021-02-22

**Authors:** Jae-Seung Yun, Yong-Moon Park, Kyungdo Han, Hyung-Wook Kim, Seon-Ah Cha, Yu-Bae Ahn, Seung-Hyun Ko

**Affiliations:** 1 Division of Endocrinology and Metabolism, Department of Internal Medicine, St. Vincent’s Hospital, College of Medicine, The Catholic University of Korea, Seoul, Korea; 2 Department of Epidemiology, Fay W. Boozman College of Public Health, University of Arkansas for Medical Sciences, Little Rock, AR USA; 3 Department of Preventive Medicine, College of Medicine, The Catholic University of Korea, Seoul, Korea; 4 Division of Nephrology, Department of Internal Medicine, St. Vincent’s Hospital, College of Medicine, The Catholic University of Korea, Seoul, Korea

**Keywords:** Endocrine system and metabolic diseases, Endocrinology, Risk factors

## Abstract

We investigated the association between the incidence of severe hypoglycemia and the risk of end-stage renal disease (ESRD) in patients with type 2 diabetes. Baseline and follow-up data for 988,333 participants with type 2 diabetes were retrieved from the National Health Insurance System database. The number of severe hypoglycemia episodes experienced from 2007 to 2009 was determined. The primary outcome was the development of ESRD after the baseline evaluation. Participants were followed from the baseline until death or December 31, 2016, during this period 14,545 participants (1.5%) developed ESRD. In the crude model, compared with those who experienced no severe hypoglycemia, the hazard ratios (95% confidential intervals) for developing ESRD were 4.96 (4.57–5.39), 6.84 (5.62–8.32), and 9.51 (7.14–12.66) in participants who experienced one, two, and three or more episodes of severe hypoglycemia, respectively. Further adjustment for various confounding factors attenuated the association between severe hypoglycemia and ESRD; the significance of the association between severe hypoglycemia and ESRD was maintained. Having three or more severe hypoglycemia episodes was associated with a nearly two-fold increased risk of developing ESRD. Prior episodes of severe hypoglycemia were associated with an increased risk of ESRD among Korean adults with type 2 diabetes.

## Introduction

Diabetes mellitus is the most common cause of end-stage renal disease (ESRD), and patients with diabetic kidney disease (DKD) account for 45% of the ESRD cases in Korea^1^. DKD contributes to the high risk of progression to ESRD and increased cardiovascular morbidity and mortality^2^. Thus, it is important to identify patients who are at risk of DKD and ESRD and initiate appropriate reno-protective treatment. Many clinical randomized controlled trials (RCTs) have demonstrated that microvascular diseases can be alleviated in patients with diabetes with intensive glycemic treatment^3–5^. A recent meta-analysis of individual participant data from RCTs has demonstrated that intensive glucose control over 5 years in patients with type 2 diabetes significantly reduced the number of renal events (mainly owing to a decrease in micro- and macroalbuminuria)^6^. Based on previous studies, clinical practice guidelines have emphasized the importance of intensive glycemic control to prevent the progression of DKD^7,8^.

However, hypoglycemia, which can occur during intensive diabetes care, has become an important health and drug safety concern^9^. Severe hypoglycemia (SH), which is defined as an event requiring the assistance of another person for treatment, is now recognized as one of the most serious adverse events in patients with type 2 diabetes, particularly among older patients and in those with renal insufficiency^10^. The association between SH, which can be caused by intensive glycemic control, and the subsequent risk of progression of DKD is unclear. While many previous studies have focused on the increased risk of hypoglycemia in patients with renal insufficiency, only a few have evaluated SH as a predictive factor for the progression of renal disease in type 2 diabetes.

Our study, therefore, aimed to determine the association between SH and the development of ESRD using data retrieved from the National Health Insurance Service (NHIS) database. We also examined the dose–response, temporal relationships, and consistency of the association between the frequency of SH events and renal outcomes.

## Results

The mean follow-up duration for the study was 6.7 years. The mean age was 61.5 ± 10.4 years, and 540,772 (54.7%) were men. At baseline, 9653 (1.0%) experienced one episode of SH, 1838 (0.2%) had at least two or more episodes of SH, and 152,270 (15.4%) had an estimated glomerular filtration rate (eGFR) < 60 mL/min/1.73 m^2^. Participants who had SH at baseline were more likely to be older, women, rural area dwellers, and insulin users. Compared with the no SH group, participants who reported having episodes of SH, included a lower proportion of current smokers, heavy drinkers, and physically active individuals and a higher proportion of those with hypertension and major comorbidities. The baseline eGFR levels tended to be lower in participants who experienced more episodes of SH (Table 1).

**Table 1:** Baseline characteristics according to the number of severe hypoglycemia event.

	Total	Number of severe hypoglycemia at baseline				*P *value
		0	1	2	3 or more	
Total	988,333	976,842 (98.8)	9653 (1.0)	1335 (0.1)	503 (0.1)	
Age (years)						< 0.0001
30–39	136,985 (13.9)	136,467 (14.0)	443 (4.6)	54 (4.0)	21 (4.2)	
40–64	456,476 (46.2)	453,419 (46.4)	2665 (27.6)	280 (21.0)	112 (22.3)	
65–	394,872 (40.0)	386,956 (39.6)	6545 (67.8)	1001 (75.0)	370 (73.6)	
Sex (male)	540,772 (54.7)	535,333 (54.8)	4555 (47.2)	624 (46.7)	260 (51.7)	< 0.0001
Diabetes duration > 5 years	526,112 (53.2)	517,703 (53.0)	7015 (72.7)	1005 (75.3)	389 (77.3)	< 0.0001
Urban	439,935 (44.5)	435,879 (44.6)	3450 (35.7)	430 (32.2)	176 (35.0)	< 0.0001
Medication						
Insulin	127,830 (12.9)	123,304 (12.6)	3628 (37.6)	616 (46.1)	282 (56.1)	< 0.0001
Sulfonylurea	772,443 (78.2)	763,449 (78.2)	7616 (78.9)	1022 (76.6)	356 (70.8)	< 0.0001
Metformin	707,468 (71.6)	699,352 (71.6)	6847 (70.9)	941 (70.5)	328 (65.2)	0.005
Meglitinide	45,813 (4.6)	44,732 (4.7)	861 (8.9)	161 (12.1)	59 (11.7)	< 0.0001
Thiazolidinedione	132,496 (13.4)	130,845 (13.4)	1395 (14.5)	189 (14.2)	67 (13.3)	0.02
DPP4 inhibitor	89,110 (9.0)	88,088 (9.0)	864 (9.0)	114 (8.5)	44 (8.8)	0.926
Acarbose	219,360 (22.2)	215,263 (22.0)	3361 (34.8)	547 (41.0)	189 (37.6)	< 0.0001
Current smoker	198,153 (20.1)	196,367 (20.1)	1479 (15.3)	212 (15.9)	95 (18.9)	< 0.0001
Heavy drinker	63,852 (6.5)	63,341 (6.5)	418 (4.3)	63 (4.7)	30 (6.0)	< 0.0001
Regular exercise	468,550 (47.4)	464,784 (47.6)	3258 (33.8)	386 (28.9)	122 (24.3)	< 0.0001
Socioeconomic status						0.065
Lower 30%	292,102 (29.6)	288,677 (29.6)	2864 (29.8)	402 (30.1)	159 (31.6)	
Mid 40%	364,539 (36.9)	360,451 (36.9)	3459 (35.8)	456 (34.2)	173 (34.4)	
Upper 30%	331,692 (33.6)	327,714 (33.6)	3330 (34.5)	477 (35.7)	171 (34.0)	
Estimated GFR (mL/min/1.73 m^2^)	79.1 ± 20.4	79.3 ± 20.3	66.7 ± 23.1	62.2 ± 23.0	60.5 ± 22.9	< 0.0001
Estimated GFR < 60 mL/min/1.73 m^2^	152,270 (15.4)	147,671 (15.1)	3710 (38.4)	631 (47.3)	258 (51.3)	< 0.0001
BMI (kg/m^2^)	25.0 ± 3.2	25.0 ± 3.2	23.9 ± 3.4	23.2 ± 3.5	22.5 ± 3.4	< 0.0001
Hypertension	648,028 (65.6)	639,180 (65.4)	7415 (76.8)	1047 (78.4)	386 (76.7)	< 0.0001
SBP (mmHg)	128.9 ± 15.6	128.9 ± 15.6	129.3 ± 16.9	129.0 ± 17.8	127.8 ± 18.1	0.01
DBP (mmHg)	78.1 ± 9.9	78.1 ± 9.9	77.1 ± 10.4	76.7 ± 10.7	76.3 ± 10.5	< 0.0001
Fasting glucose (mmol/L)	7.79 ± 2.63	7.79 ± 2.62	7.40 ± 2.98	7.39 ± 3.16	7.91 ± 3.54	< 0.0001
Total cholesterol (mmol/L)	4.91 ± 1.05	4.91 ± 1.05	4.73 ± 1.09	4.65 ± 1.11	4.68 ± 1.11	< 0.0001
Triglyceride (mmol/L)	1.61 (1.61–1.61)	1.61 (1.61–1.61)	1.50 (1.49–1.52)	1.43 (1.39–1.48)	1.40 (1.34–1.47)	< 0.0001
HDL-cholesterol (mmol/L)	1.32 ± 0.51	1.32 ± 0.51	1.30 ± 0.55	1.31 ± 0.61	1.35 ± 0.65	0.047
LDL-cholesterol (mmol/L)	2.77 ± 1.13	2.77 ± 1.13	2.68 ± 1.22	2.60 ± 0.99	2.62 ± 1.06	< 0.0001
Dyslipidemia	479,798 (48.6)	474,547 (48.6)	4478 (46.5)	575 (43.2)	198 (39.6)	< 0.0001
Cardiovascular disease	78,291 (7.9)	75,949 (7.8)	1879 (19.5)	322 (24.1)	141 (28.0)	< 0.0001
Stroke	64,962 (6.6)	63,008 (6.5)	1562 (16.2)	268 (20.1)	124 (24.7)	< 0.0001
COPD	33,313 (3.4)	32,848 (3.4)	392 (4.1)	54 (4.0)	19 (3.8)	0.001
Cancer	9009 (0.9)	8841 (0.9)	145 (1.5)	16 (1.2)	7 (1.4)	< 0.0001
Liver cirrhosis	2286 (0.2)	2248 (0.2)	30 (0.3)	5 (0.4)	3 (0.6)	0.079
Major comorbidities	117,205 (11.9)	114,388 (11.7)	2283 (23.7)	375 (28.1)	159 (31.6)	< 0.0001

Values are presented as n (%) or percentage or mean ± standard deviation.

*DPP4* dipeptidylpeptidase 4, *GFR* glomerular filtration rate, *SBP* systolic blood pressure, *DBP* diastolic blood pressure, *COPD* chronic obstructive pulmonary disease.

**Figure 1:**
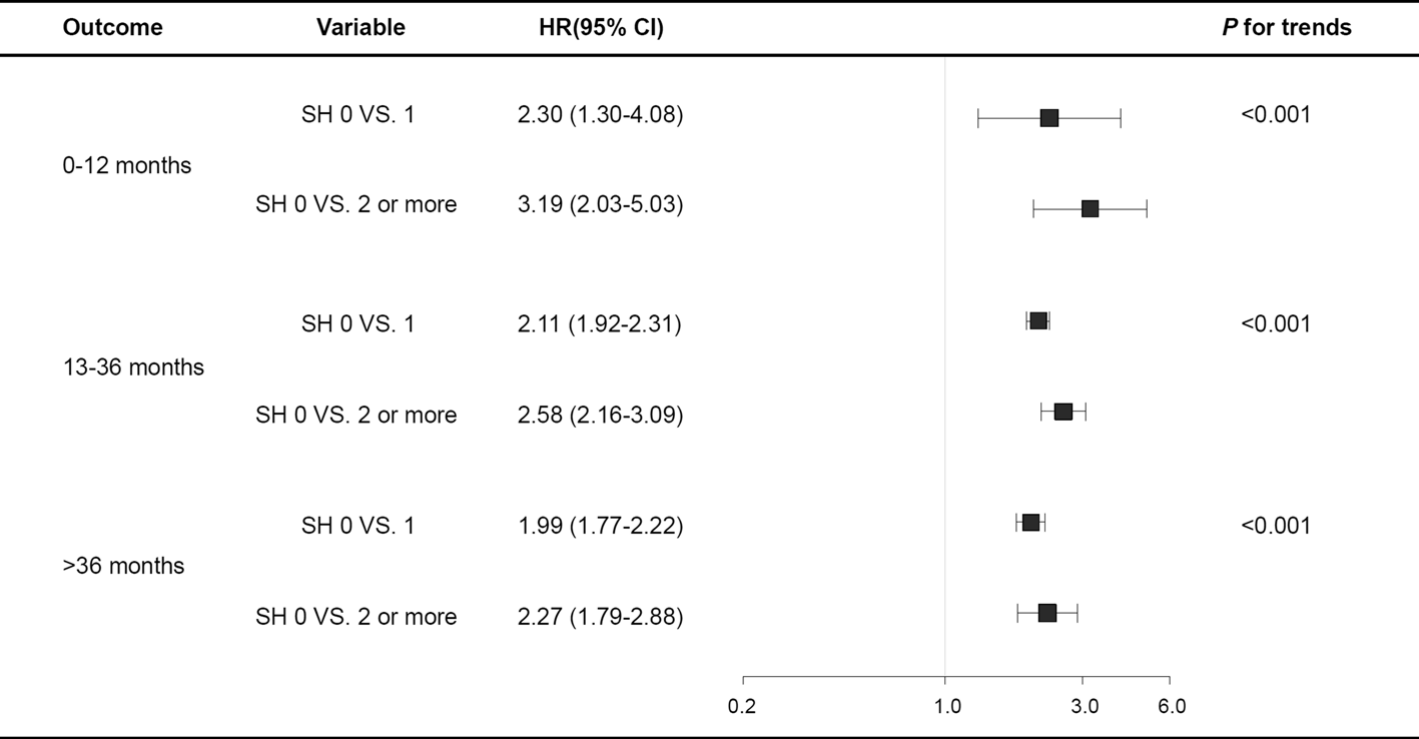
The association between severe hypoglycemia and outcomes by time since index date. All HRs adjusted for covariates including age, sex, smoking, alcohol consumption, regular exercise, living place (urban or rural), income level, BMI, hypertension, dyslipidemia, chronic kidney disease, urine protein, anti-diabetic drugs, diabetes duration, fasting plasma glucose, LDL-cholesterol, and major comorbidities (malignancy, liver cirrhosis, and chronic obstructive pulmonary disease).

During the follow-up period, 14,545 participants (1.5%) developed ESRD, and the incidence of ESRD was 2.2 per 1000 patients-years. In the crude model, compared with those who experienced no SH, the hazard ratios [HRs] (95% confidential intervals) for developing ESRD were 4.96 (4.57–5.39), 6.84 (5.62–8.32), and 9.51 (7.14–12.66) in participants who experienced one, two, and three or more episodes of SH, respectively (Table 2). Although further adjustment for confounding factors, including the eGFR status, proteinuria, use of insulin, sulfonylurea, ACE inhibitor and ARBs, fasting glucose levels, and major comorbidities, attenuated the associations between SH and the incidence of ESRD, this readjustment did not change the significance of the main effects. The renal outcomes observed showed a dose–response relationship with the number of SH episodes experienced. The presence of three or more SH episodes was associated with a nearly two-fold increased risk of developing ESRD, and the HRs showed an increasing trend as the number of SH episodes increased (Table 2). All HRs for ESRD were higher in the group with an SH event within 1 year than in the group with an SH event 1 year from the index date (Fig. 1).

**Table 2:** Crude and adjusted HRs of end-stage renal disease.

Number of SH	N	Event	Incidence rate	HR (95% CI)		
			(per 1000 person-years)	Model 1	Model 2	Model 3
0	976,842	13,812	2.11	Reference	Reference	Reference
1	9653	585	10.23	4.96 (4.57–5.39)	4.54 (4.18–4.93)	1.71 (1.57–1.86)
2	1335	101	13.85	6.84 (5.62–8.32)	6.09 (5.01–7.41)	1.78 (1.46–2.18)
3 or more	503	47	18.9	9.51 (7.14–12.66)	8.24 (6.18–10.97)	1.86 (1.39–2.48)
*P* for trends				< 0.001	< 0.001	< 0.001

Model 1: Unadjusted.

Model 2: Adjusted for age, sex.

Model 3: Model 2+ smoking, alcohol consumption, regular exercise, living place (urban or rural), income level, BMI, hypertension, dyslipidemia, chronic kidney disease, urine protein, anti-diabetic drugs (biguanide, sulfonylurea, a-glucosidase inhibitor, thiazolidinedione, meglitinide, dipeptidyl peptidase-4 inhibitor, and insulin), ACE inhibitor/ARBs, diabetes duration > 5 years, fasting plasma glucose, LDL-cholesterol, and major comorbidities (cardiovascular disease, malignancy, liver cirrhosis, and COPD).

*SH* severe hypoglycemia, *ARB* angiotensin receptor blocker, *COPD* chronic obstructive pulmonary disease.

**Figure 2:**
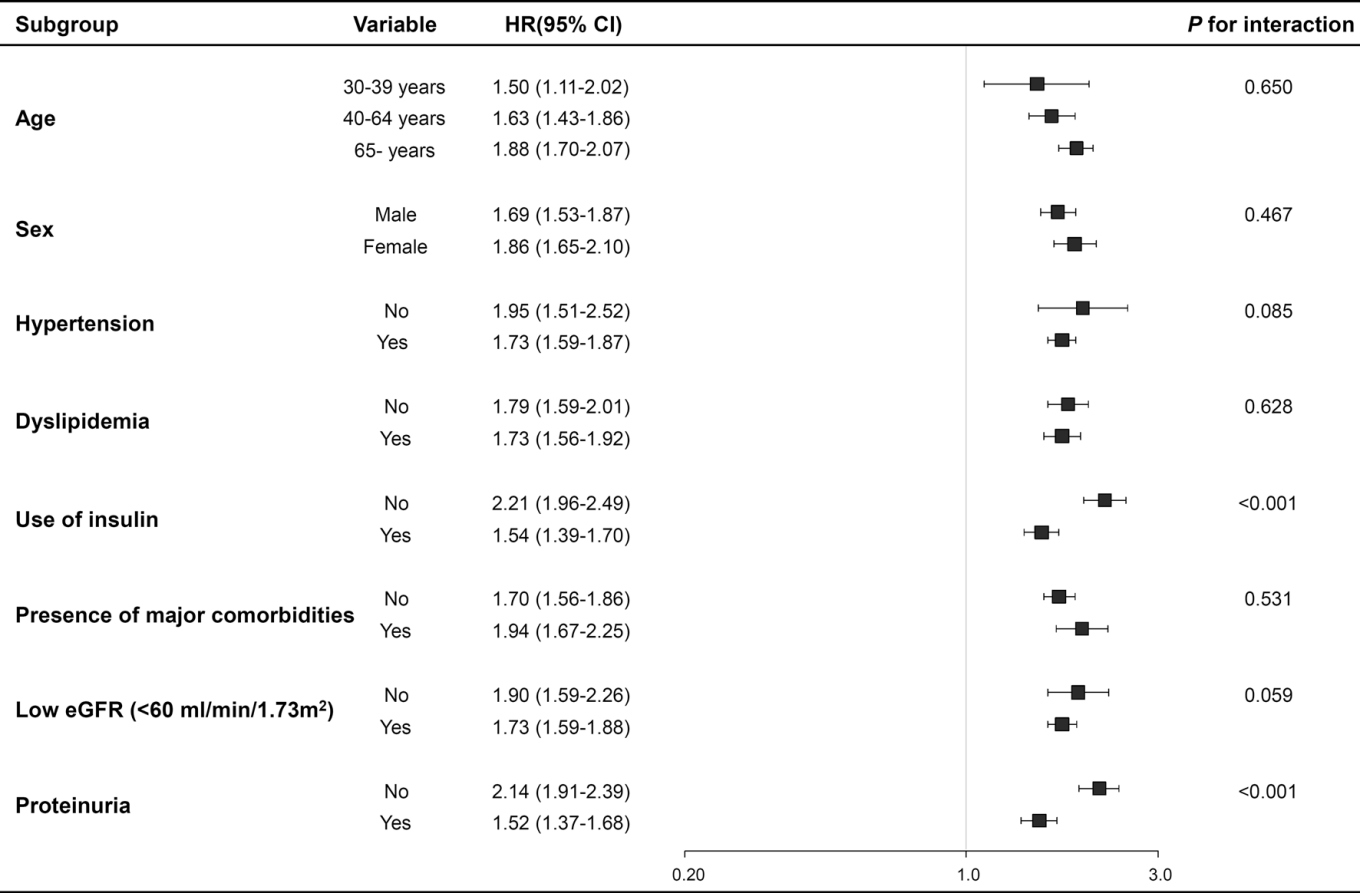
Subgroup analysis for the association between recurrent severe hypoglycemia and end-stage renal disease. All HRs adjusted for covariates including age, sex, smoking, alcohol consumption, regular exercise, living place (urban or rural), income level, BMI, hypertension, dyslipidemia, chronic kidney disease, urine protein, anti-diabetic drugs, diabetes duration, fasting plasma glucose, LDL-cholesterol, and major comorbidities (malignancy, liver cirrhosis, and chronic obstructive pulmonary disease).

In the participants with proteinuria, the risk of developing ESRD was about two times higher in participants with a history of SH than in those with no history of SH. Moreover, the risk of ESRD increased by eleven times in participants with a history of both proteinuria and SH than in participants without proteinuria and a history of SH (*P* for interaction < 0.001). Similarly, in the participants with an eGFR < 60 mL/min/1.73 m^2^, the risk of ESRD was doubled in participants with a history of SH compared with those with no history of SH. The risk of ESRD increased by 12.8 times in participants with a history of both low eGFR and SH (*P* for interaction = 0.06, Supplementary Figure S1). We also performed subgroup analyses based on the stratification by age, the presence of hypertension, dyslipidemia, use of insulin, and major comorbidities. The results revealed no changes in the main relationships between SH episodes and subsequent ESRD events. No interactions with sex, age, hypertension, or dyslipidemia were observed in the relationship, except for renal insufficiency and insulin use (Fig. 2). The results of the sensitivity analyses after further adjusting the covariates for the additional modified follow-up time from the occurrence of SH to the end of the follow-up period and excluding the participants who experienced SH events during the follow-up period did not change the significance of the association between SH events and ESRD (Supplementary Table S1 and Supplementary Table S2). To balance the differences in baseline characteristics, we performed a subgroup analysis (n = 22,442) with propensity score matching in the sensitivity analysis. After matching for multiple confounding factors, there were no significant differences between the two groups (Supplementary Table S3). The dose-dependent relationship between SH and ESRD was confirmed using Cox regression analysis with propensity score matching data (Supplementary Table S4).

## Discussion

To the best of our knowledge, this is the first study to investigate the dose–response relationship, temporal relationship, and the consistency of the relationship between SH and renal outcomes. Our findings demonstrate an association between episodes of SH and an increased risk of renal outcomes. We found a dose–response, temporal relationship between SH and renal outcomes, wherein the participants who experienced more SH events were at a higher risk of developing ESRD. These results remained consistent in the multiple subgroups regardless of the presence of high-risk factors for renal disease progression as well as in the subgroup after propensity score matching of the major baseline characteristics affecting renal function.

Previous studies have shown that poor glycemic control increases the incidence of microvascular complications^11–15^. Thus, intensive glycemic control is believed to reduce the progression of DKD. Previous clinical trials demonstrated that intensive glycemic control prevented the onset of micro- and macroalbuminuria, which could account for its reno-protective effects^3,16,17^. However, except for the ADVANCE trial, the findings of other trials including the UKPDS, ACCORD, VADT as well as a meta-analysis of these studies have failed to demonstrate the protective effects of intensive glycemic control on the risk of doubling the serum creatinine levels or developing ESRD^18^. The suggested reasons for the ineffectiveness of intensive glycemic control in preventing renal outcomes included a short study duration to evaluate the overt improvement in progressive DKD, insufficient magnitude of HbA1c reduction for having an impact on clinical renal outcomes, attenuation of the preventive effects when the glycemic levels reached a certain low in a specific group (ceiling effect), and attenuation of the preventive effects on renal outcomes owing to the increased risk of SH. Intensive glycemic control significantly increased the risk of SH compared with conventional glycemic control. Owing to the heterogeneity in the characteristics of study populations and glycemic targets, these studies could not clearly conclude whether intensive glycemic control inhibits the progression of DKD despite the greater risk of SH. There are few previous studies assessing whether hypoglycemia or SH may affect the decline in renal function. A retrospective observational study from Taiwan demonstrated that SH is associated with kidney dysfunction at the 8-month follow-up in type 2 diabetes^19^. Other studies examined the association between SH and microvascular complications including renal manifestations^20,21^. Recently, a study was published using data from the Taiwan NHI database, and the authors suggested an association between SH and ESRD^22^. However, this study did not adjust for various confounding factors such as levels of fasting plasma glucose or LDL-cholesterol, and furthermore, their study did not perform sensitivity analyses in the various subgroups.

While the exact mechanism between prior SH episodes and renal outcomes is not well understood, some potential relationships, such as inflammation, defects in coagulation, endothelial dysfunction, or abnormal sympathoadrenal responses^11,23–27^, can be proposed between the two in patients with diabetes. One Japanese study reported that the immediate surge of blood pressure after an SH event can accelerate the deterioration of renal function^28^. Thus, hypoglycemia may increase the risk of DKD progression and ESRD. The current ADA guidelines recommend intensive glycemic control to prevent DKD and a raised glycemic target (HbA1c over 8%, 64 mmol/mol) for patients with a low eGFR who are at risk of hypoglycemia^29^. However, raising the glycemic target for patients with a low eGFR is a recommendation that only considers the increased risk of hypoglycemia and not the effects of hypoglycemia with the progression of renal disease.

Although the analyses of the whole cohort as well as the subgroups indicate an association between SH episodes and the subsequent development of ESRD, the results of this study do not confirm that SH can be a direct cause of ESRD. Other factors such as a higher glycemic variability and long-term diabetes, which are common in patients who experience episodes of SH, were not considered in this analysis and might have had an indirect effect on the progression of renal disease. In general, the patients who are at a high risk of SH are in the advanced stages of diabetes and may be more vulnerable to renal damage caused by SH^10^. To overcome reverse causation bias by confounding factors of the SH study, we conducted an additional analysis to demonstrate the dose–response relationship, temporal relationship, and consistency of the relationship between SH and renal outcomes. In our real-world analysis, there was a clear dose–response and temporal relationship between the frequency of SH events and ESRD. The findings of this study remained significant even after adjusting for several potential confounders, such as glycemic status, antidiabetic medications, and major comorbidities, all of which can affect renal outcomes significantly. It is not easy to demonstrate the direct causal relationship between SH and ESRD clearly. The best way to prove the causal relationship in an intervention study. As the observation of outcomes after inducing SH is ethically impossible, we identified factors that indirectly suggest causality, such as dose–response and temporality. The dose–response and temporal relationship between SH episodes and the incidence of ESRD also suggest the possibility of a direct causal linkage between SH and ESRD^30^. Additionally, the findings remained unchanged in the subgroup analyses, which included participants with an eGFR ≥ 60 mL/min/1.73 m^2^, proteinuria, and major comorbidities as well as those who did not use insulin, and even in the subgroup analyses after propensity matching of the subgroups.

Patients with type 2 diabetes and renal insufficiency tend to have an increased risk of hypoglycemia. In our analysis, the participants who experienced an SH event with renal insufficiency (in this study, eGFR < 60 mL/min/1.73 m^2^ or proteinuria) were 11–12 times more likely to progress to ESRD than participants without an SH experience and renal insufficiency. There were effect modifications from renal insufficiency and insulin use on the relationship between SH and subsequent ESRD events. The effect of SH on renal progression was attenuated in the participants who had renal insufficiency or used insulin, owing to the possibility that the group of patients with these conditions may have included more patients with high-risk factors for progression of renal function, including longer diabetes duration, older age, comorbidities, or renal insufficiency itself.

Our study has certain limitations. First, it was based on a medical claims database. Therefore, the discrepancies between the diagnosis submitted in the claims and in real medical practice could have potentially resulted in misclassifications of the disease status. To address this limitation, we combined the laboratory data with information obtained through a standardized questionnaire. Second, our study relied on the medical claims and National Health checkup data, which lacked information on important factors that affect renal outcomes in diabetes, such as duration of the disease, HbA1c levels, and use of medications and their doses. We performed several sensitivity analyses to determine possible bias, including selection, misclassification, and immortal time bias. However, exclusion of patients with previous ESRD or death events prior to the start of this study may have contributed to under- or overestimation of the SH-ESRD relationship. In addition, we performed the analysis using National Health checkup data and included only participants who had undergone a national health examination. Therefore, there is a possibility of selection bias, and patients with severe disabilities would likely have been excluded. Thus, additional standardized, high-quality community or hospital-based cohort studies are needed. Despite these limitations, the strength of this study is that we analyzed long-term follow-up longitudinal data of the entire Korean population.

In summary, the findings of this study demonstrated the potential prognostic importance of SH on the progression of renal disease. Our observational study indicated that recurrent SH showed a strong and positive association with the subsequent development of ESRD. Therefore, in patients who are at risk of recurrent hypoglycemia, it is important to prevent episodes of SH to avoid the progression of renal disease. Setting appropriate individualized glycemic targets for high-risk SH patients, careful management, and frequent monitoring of all individuals with type 2 diabetes, are strategies that can potentially help minimize the risk of hypoglycemia. However, further studies are needed to clarify whether SH is the cause or solely a marker in renal disease progression.

## Methods

### Data source and study population

This study analyzed data from the nationally representative Korean National Health Insurance (NHI) Claims Database. The National Health Insurance Corporation, a government agency that administers the medical service system in Korea^31^, is a single insurer managed by the Korean government. The NHIS is an obligatory system, and approximately 97% of the Korean citizens are insured with this system. All Korean adults aged > 30 years are encouraged to undergo a biennial, standardized medical examination, which includes measurements of weight and height as well as laboratory tests including fasting blood glucose, lipid profiles, liver enzymes, urinalysis, and eGFR. In addition, a questionnaire on smoking, alcohol consumption status, and physical activity is administered.

In this study, the participants were followed from the date of collection of the baseline information until death or December 31, 2016, whichever came earlier. In total, 2,061,659 participants aged ≥ 30 years who were diagnosed with type 2 diabetes based on ICD-10 codes in 2009 were included in this study. Type 2 diabetes was defined as having fasting plasma glucose levels (≥ 7.0 mmol/L) or clinical diagnostic codes (ICD-10 codes: E11–E14) and being prescribed anti-diabetic drugs. After applying the inclusion and exclusion criteria, 988,333 participants were finally included in the study (Supplementary Figure S2). To protect the privacy of the participants, all information was anonymized. Patient-informed consent was not required by the Catholic Medical Center of Korea Institutional Review Board owing to the retrospective nature of the study. This study was approved by the Catholic Medical Center of Korea Institutional Review Board (approval number: VC19ZESI0004) and was conducted in compliance with the guidelines of the Declaration of Helsinki.

### Definition of variables and measurements

SH was defined using the diagnostic clinical codes for hypoglycemia [International Classification of Disease, 10th (ICD-10) codes: E 16.x, E1163, E1363, and E1463). Owing to limitations in the claims data, we evaluated all the reported episodes of SH from the inpatient or emergency room claims dataset and counted the number of cases enrolled between January 1, 2007, and December 31, 2009. For the enrolled cases, the index date was set at January, 2010. The primary endpoint for this study was incident ESRD, which was defined as patients who had made a claim using the ICD-10 codes (N18, N19, Z49, Z94.0, and Z99.2), as well as the procedure code of renal replacement therapy and/or kidney transplantation (R380, O7011–7020, O7017, and O7075) during the follow-up period. The methodology for defining ESRD has been described in detail elsewhere^32^.

Body mass index (BMI) was calculated as weight in kilograms divided by the square of height in meters (kg/m^2^). The definition of urban and rural regions was based on the administrative district of Korea. Socioeconomic status was categorized into lower 30%, mid 40%, and upper 30% groups according to their income levels. Information on current smoking, alcohol consumption, and regular exercise was collected using a standard questionnaire. Anti-diabetic drugs included the seven classes (insulin, sulfonylureas, biguanides, meglitinides, thiazolidinediones, dipeptidyl peptidase-4 inhibitors, and a-glucosidase inhibitors) that were available in Korea at the time when the baseline indices for the study were being determined.

Cardiovascular disease, all types of cancer, liver cirrhosis, and chronic obstructive pulmonary disease were selected as the major serious comorbidities to adjust the possibility of reverse causation bias. Comorbidities as covariates were defined using ICD-10 codes. These are summarized in Supplementary Table S5. The eGFR was calculated using the Modification of Diet in the Renal Disease formula. An eGFR < 60 mL/min/1.73 m^2^ was considered low. The level of protein in fresh, midstream urine samples was measured semi-quantitatively using a urine dipstick. Proteinuria was diagnosed based on a reading of 1+ or greater with the urine dipstick test. Hospitals, where the national health examinations were performed, were subjected to regular quality control assessments and certified by the NHIS.

### Statistical analysis

Categorical variables are expressed as numbers (%) and continuous variables as means ± SD. The characteristics of the groups based on the number of SH events were compared using a one-way analysis of variance or the chi-squared test. We evaluated the association between the number of SH events with incident ESRD using Cox proportional hazards regression analysis. HRs and 95% confidence intervals were used to evaluate the association between the SH episodes and renal outcomes in Cox proportional hazards models. We also adjusted the models for potential confounders, which were determined using both statistical methods and findings of previous studies. Death during the follow-up period was considered as a competing risk factor for ESRD, and cases involving death during the follow-up period (n = 86,716) were excluded from Cox regression analysis. We conducted subgroup analyses stratified by the confounding factors for the sensitivity analysis. The potential effect of modifications by the subgroups was evaluated using stratified analysis and interaction testing with a likelihood ratio test. To avoid immortal time bias, we performed an additional analysis that adjusted the covariates for the additional modified follow-up time (from the actual occurrence of SH to the end of the follow-up period in the SH group). In addition, we performed Cox proportional hazard regression analysis after excluding the participants with SH during the follow-up period.

Propensity score matching was conducted as an additional method to adjust for differences in the clinical characteristics between the groups with and without SH. The propensity scores were estimated using SAS (SAS Institute, USA) using the matching macro, “%OneToManyMTCH”, with 1:1 matching. These were adjusted for all the confounding variables that were used in the main model as covariates. Differences between groups after matching for each variable were examined using the chi-square test and the independent t-test to determine which variables were adequately matched. We then performed Cox regression analysis in the matched cohort. All statistical analyses were performed using the Statistical Package SAS 9.4, and *P* values < 0.05 were considered significant.

## Supplementary Information

Supplementary Information
