# Supplementary material for: Severe hypoglycemia and the risk of end stage renal disease in type 2 diabetes
Source: Sci Rep. 2021 Feb 22;11:4305. doi: 10.1038/s41598-021-82838-5 (PMC7900096; doi:10.1038/s41598-021-82838-5)
Supplement: Supplementary file 1 — Supplementary Information [file 41598_2021_82838_MOESM1_ESM.docx]

**Severe Hypoglycemia and the Risk of End Stage Renal Disease in Type 2 Diabetes**

**Running Title: Severe hypoglycemia and ESRD in type 2 diabetes**

Jae-Seung Yun, MD, PhD^1^, Yong-Moon Park, MD, PhD^2^, Kyungdo Han MD, PhD^3^, Hyung-Wook Kim, MD, PhD^4^, Seon-Ah Cha MD, PhD^1^, Yu-Bae Ahn, MD, PhD^1^, Seung-Hyun Ko, MD, PhD^1^

^1^Division of Endocrinology and Metabolism, Department of Internal Medicine, St. Vincent’s Hospital, College of Medicine, The Catholic University of Korea, Seoul, Korea

^2^Epidemiology Branch, National Institute of Environmental Health Sciences, National Institutes of Health, Research Triangle Park, NC, USA

^3^Department of Preventive Medicine, College of Medicine, The Catholic University of Korea, Seoul, Korea

^4^Division of Nephrology, Department of Internal Medicine, St. Vincent’s Hospital, College of Medicine, The Catholic University of Korea, Seoul, Korea

**Corresponding Authors:** **Seung-Hyun Ko, MD, PhD**

Professor

Division of Endocrinology and Metabolism

Department of Internal Medicine

St. Vincent’s Hospital

College of Medicine, The Catholic University of Korea

Seoul, Korea

**E-mail: kosh@catholic.ac.kr**

Supplementary Table 1. Cox regression analysis after adjusting immortal time

| Number of SH | N | Event | Incidence rate | HR (95% CI) | | | |
| --- | --- | --- | --- | --- | --- | --- | --- |
|  |  |  | (per 1000 person-years) | Model 1 | Model 2 | Model 3 | Model 4 |
| 0 | 976,842 | 13,812 | 2.11 | Reference | Reference | Reference | Reference |
| 1 | 9,653 | 585 | 10.23 | 4.96 (4.57-5.39) | 4.54 (4.18-4.93) | 1.71 (1.57-1.86) | 2.09 (1.82-2.40) |
| 2 | 1,335 | 101 | 13.85 | 6.84 (5.62-8.32) | 6.09 (5.01-7.41) | 1.78 (1.46-2.18) | 2.12 (1.70-2.63) |
| 3 or more | 503 | 47 | 18.9 | 9.51 (7.14-12.66) | 8.24 (6.18-10.97) | 1.86 (1.39-2.48) | 2.22 (1.65-2.97) |
| *P* for trends |  |  |  | <0.001 | <0.001 | <0.001 | <0.001 |

SH, Severe hypoglycemia; ARB, angiotensin receptor blocker; COPD, chronic obstructive pulmonary disease

Model 1: Unadjusted

Model 2: Adjusted for age, sex

Model 3: Model 2 + smoking, alcohol consumption, regular exercise, living place (urban or rural), income level, BMI, hypertension, dyslipidemia, chronic kidney disease, urine protein, anti-diabetic drugs (biguanide, sulfonylurea, a-glucosidase inhibitor, thiazolidinedione, meglitinide, dipeptidyl peptidase-4 inhibitor, and insulin), ACE inhibitor/ARBs, diabetes duration > 5 years, fasting plasma glucose, LDL-cholesterol, and major comorbidities (cardiovascular disease, malignancy, liver cirrhosis, and COPD).

Model 4: Model 3 + modified follow-up time

Supplementary Table 2. Time dependent Cox regression analysis from the sub-cohort with exclusion of subjects who experienced SH during the follow-up period

| Number of SH | HR (95% CI) | | |
| --- | --- | --- | --- |
|  | Model 1 | Model 2 | Model 3 |
| 0 | Reference | Reference | Reference |
| 1 | 4.72 (4.35-5.12) | 4.28 (3.94-4.65) | 1.58 (1.45-1.72) |
| 2 or more | 7.60 (6.56-8.80) | 6.65 (5.73-7.70) | 1.76 (1.51-2.04) |
| *P* for trends | <0.001 | <0.001 | <0.001 |

SH, Severe hypoglycemia; ARB, angiotensin receptor blocker; COPD, chronic obstructive pulmonary disease

Model 1: Unadjusted

Model 2: Adjusted for age, sex

Model 3: Model 2 + smoking, alcohol consumption, regular exercise, living place (urban or rural), income level, BMI, hypertension, dyslipidemia, chronic kidney disease, urine protein, anti-diabetic drugs (biguanide, sulfonylurea, a-glucosidase inhibitor, thiazolidinedione, meglitinide, dipeptidyl peptidase-4 inhibitor, and insulin), ACEi/ARB, diabetes duration > 5 years, fasting plasma glucose, LDL-cholesterol, and major comorbidities (cardiovascular disease, malignancy, liver cirrhosis, and COPD).

Supplementary Table 3. Baseline characteristics between groups after propensity score matching

|  | SH (-) | SH (+) | *P-*value |
| --- | --- | --- | --- |
| Number | 11,221 | 11,221 |  |
| Age | 68.0 ± 9.0 | 68.0 ± 9.9 | 0.722 |
| Sex (male) | 5,302 (47.3) | 5,325 (47.5) | 0.759 |
| Diabetes duration >5 years | 8,187 (73.0) | 8,205 (73.1) | 0.787 |
| Medication |  |  |  |
| Insulin | 4,369 (38.9) | 4,429 (39.5) | 0.412 |
| Sulfonylurea | 8,723 (77.7) | 8,767 (78.1) | 0.479 |
| Metformin | 7,981 (71.1) | 7,926 (70.6) | 0.419 |
| Current smoker | 1,743 (15.5) | 1,754 (15.6) | 0.840 |
| Heavy drinker | 475 (4.2) | 498 (4.4) | 0.451 |
| Regular exercise | 3,713 (33.1) | 3,712 (33.1) | 0.989 |
| Socioeconomic status |  |  | 0.335 |
| Lower 30% | 3,329 (29.7) | 3,338 (29.8) |  |
| Mid 40% | 3,905 (34.8) | 3,993 (35.6) |  |
| Upper 30% | 3,987 (35.5) | 3,890 (34.7) |  |
| Hypertension | 8,660 (77.2) | 8,644 (77.0) | 0.799 |
| Dyslipidemia | 5,240 (46.7) | 5,169 (46.1) | 0.342 |
| Fasting glucose (mmol/L) | 133.4 ± 45.6 | 133.7 ± 54.4 | 0.642 |
| BMI (kg/m^2^) | 23.8 ± 3.1 | 23.8 ± 3.5 | 0.976 |
| Low eGFR | 4,201 (37.4) | 4,240 (37.8) | 0.591 |
| Urine protein | 1,484 (13.2) | 1,559 (13.9) | 0.144 |
| Major comorbidities | 2,679 (23.9) | 2,730 (24.3) | 0.426 |

Values are presented as n (%) or percentage or mean ± standard deviation.

Supplementary Table 4. Cox regression analysis after propensity score matching

| Number of SH | N | Event | Incidence rate | HR (95% CI) |
| --- | --- | --- | --- | --- |
|  |  |  | (per 1000 person-years) |  |
| 0 | 11,221 | 440 | 6.24 | Reference |
| 1 | 9,452 | 575 | 10.24 | 1.65 (1.45-1.86) |
| 2 | 1,286 | 96 | 13.55 | 2.19 (1.75-2.73) |
| 3 or more | 483 | 47 | 19.62 | 3.18 (2.36-4.30) |

SH, Severe hypoglycemia; ARB, angiotensin receptor blocker; COPD, chronic obstructive pulmonary disease

Model: Age, sex, smoking, alcohol consumption, regular exercise, living place (urban or rural), income level, BMI, hypertension, dyslipidemia, chronic kidney disease, urine protein, anti-diabetic drugs (biguanide, sulfonylurea, a-glucosidase inhibitor, thiazolidinedione, meglitinide, dipeptidyl peptidase-4 inhibitor, and insulin), ACEi/ARB, diabetes duration > 5 years, fasting plasma glucose, LDL-cholesterol, and major comorbidities (cardiovascular disease, malignancy, liver cirrhosis, and COPD).

Supplementary Table 5. Definitions of comorbidities used as main outcome or covariates in this study

| **Variable** | **Definition** |
| --- | --- |
| Cardiovascular disease | ICD-10 codes: I21, I22, I63, I64 |
| Cancer | ICD-10 codes: C00-C97 |
| Chronic obstructive pulmonary disease | ICD-10 codes: J43, J44 |
| Dyslipidemia | (ICD-10 code: E78 and  prescription of lipid-lowering drugs) or total cholesterol ≥6.2 mmol/L |
| End-stage renal disease | ICD-10 codes: N18, N19, Z49, Z94.0, Z99.2  and Procedure code: R380, O7011-7020, O7017, O7075 |
| Hypertension | (ICD-10 code: I10-I13, I15 and  prescription of anti-hypertensive drugs) or systolic/diastolic blood pressure over 140/90 mmHg |
| Liver cirrhosis | ICD-10 codes: K704, K746 |
| Severe hypoglycemia | ICD-10 codes: E16.x, E1163, E1363, E1463 |
| Type 2 diabetes | (ICD-10 code: E11, E14 and  prescription of anti-diabetic drugs) or fasting plasma glucose over 7.0 mmol/L |


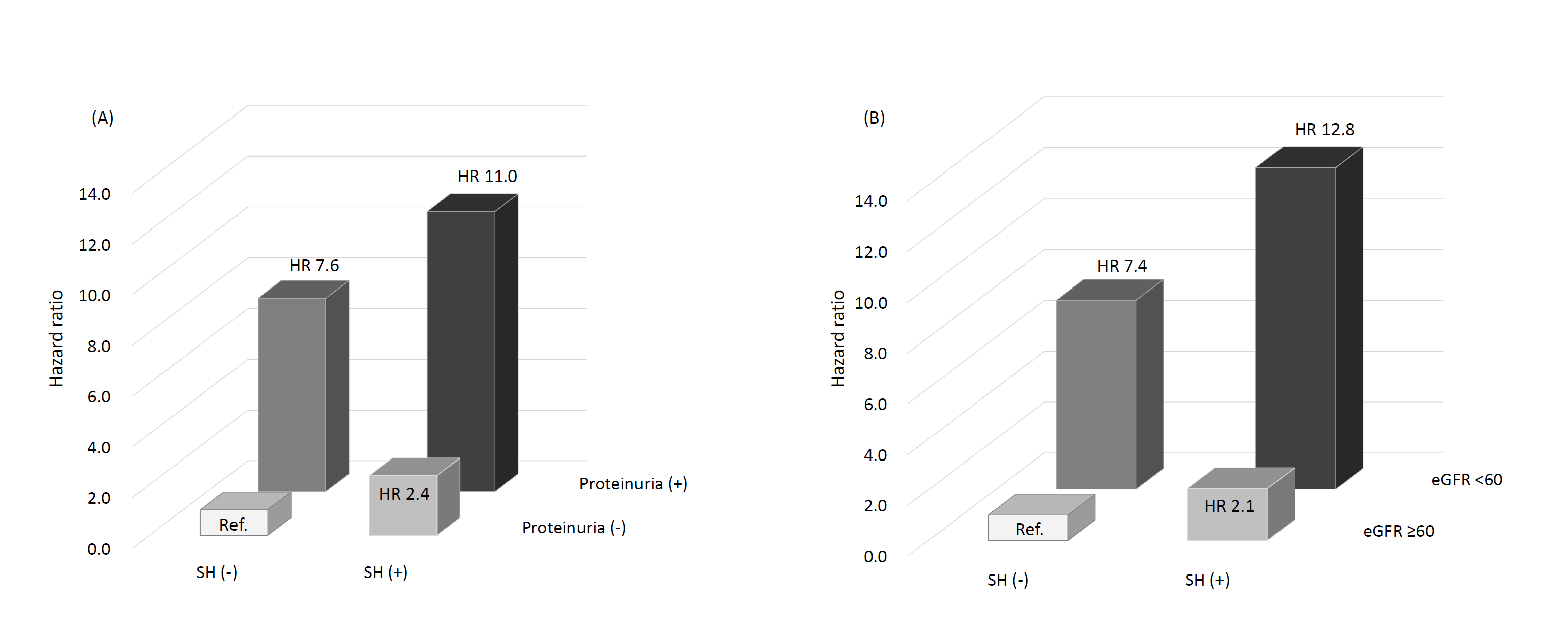


Supplementary Figure 1. The risk comparison of severe hypoglycemia for end-stage renal disease between (A) the subjects with and without proteinuria, (B) the subjects with and without low estimated glomerular filtration rate


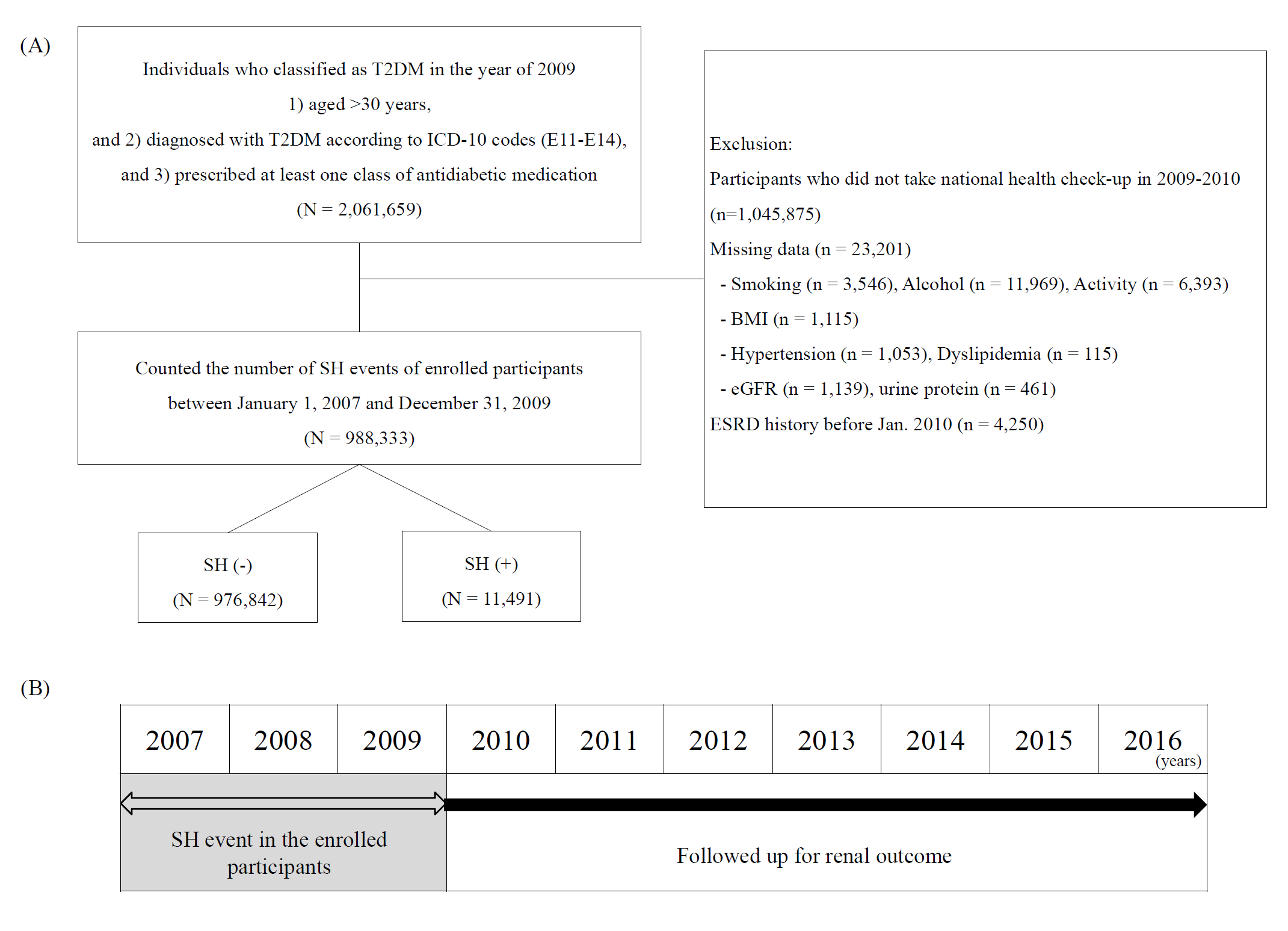


Supplementary Figure 2. (A) Sample recruitment from the database of National Health Insurance Service (B) Study design summarizing subjects and follow-up
